# Tuning Lignin Characteristics by Fractionation: A Versatile Approach Based on Solvent Extraction and Membrane-Assisted Ultrafiltration

**DOI:** 10.3390/molecules25122893

**Published:** 2020-06-23

**Authors:** Chiara Allegretti, Oussama Boumezgane, Letizia Rossato, Alberto Strini, Julien Troquet, Stefano Turri, Gianmarco Griffini, Paola D’Arrigo

**Affiliations:** 1 Department of Chemistry, Materials and Chemical Engineering “Giulio Natta”, Politecnico di Milano, p.zza L. da Vinci 32, 20133 Milano, Italy; chiara.allegretti@polimi.it (C.A.); oussama.boumezgane@polimi.it (O.B.); l.rossato1@campus.unimib.it (L.R.); stefano.turri@polimi.it (S.T.); 2 Dipartimento di Biotecnologie e Bioscienze, Università di Milano-Bicocca, Piazza della Scienza 2, 20126 Milano, Italy; 3 Istituto per le Tecnologie della Costruzione, Consiglio Nazionale delle Ricerche (ITC-CNR), via Lombardia 49, 20098 San Giuliano Milanese, Italy; alberto.strini@itc.cnr.it; 4 Biobasic Environnement, Biopôle Clermont-Limagne, 63360 Saint-Beauzire, France; jtroquet@biobasicenvironnement.com; 5 Istituto di Scienze e Tecnologie Chimiche “Giulio Natta”, Consiglio Nazionale delle Ricerche (SCITEC-CNR), via Mario Bianco 9, 20131 Milano, Italy

**Keywords:** lignin, fractionation, biobased polymers, solvent extraction, membrane-assisted ultrafiltration

## Abstract

Technical lignins, typically obtained from the biorefining of lignocellulosic raw materials, represent a highly abundant natural aromatic feedstock with high potential in a sustainable economy scenario, especially considering the huge primary production volumes and the inherently renewable nature of this resource. One of the main drawbacks in their full exploitation is their high variability and heterogeneity in terms of chemical composition and molecular weight distribution. Within this context, the availability of effective and robust fractionation processes represents a key requirement for the effective valorization of lignin. In the present work, a multistep fractionation of two different well known technical lignins obtained from two distinct delignification processes (soda vs. kraft pulping) was described. A comprehensive approach combining solvent extraction in organic or aqueous medium with membrane-assisted ultrafiltration was developed in order to maximize the process versatility. The obtained lignin fractions were thoroughly characterized in terms of their chemical, physical, thermal, and structural properties, highlighting the ability of the proposed approach to deliver consistent and reproducible fractions of well-controlled and predictable characteristics, irrespective of their biomass origin. The results of this study demonstrate the versatility and the reliability of this integrated multistep fractionation method, which can be easily adapted to different solvent media using the same ultrafiltration membrane set up, thereby enhancing the potential applicability of this approach in an industrial scale-up perspective for a large variety of starting raw lignins.

## 1. Introduction

Lignin is a biobased aromatic amorphous polymer composed of a complex methoxylated phenylpropane skeleton, which represents on average about 24% of the total components of plants [1] and, consequently, a substantial amount of the organic carbon in the biosphere [2]. It acts as a natural glue, providing the plants’ structural integrity and resistance against environmental and biological degradation [3]. Technical lignins are typically obtained from the biorefining of vegetal feedstocks for the valorization of cellulose and hemicelluloses, the so-called sugar-based platform [4,5,6]. Another major source of technical lignins is the pulp and paper manufacturing sector, where it is produced at an approximate rate of about 50 million tons per year [7]. Within these contexts, lignin is usually considered a waste byproduct with low residual value. To date, only a small part of the technical lignins produced worldwide is actually used for material manufacturing and chemical synthesis, as the majority is being burnt for energy recovery at the processing plant itself [8]. The main existing pathways for lignin valorization aim at its direct utilization (e.g., as micro or nanostructured filler in blend with polymers [9,10] or as a water-reducing agent for concrete [11]) with limited processing after the initial cellulose separation from the original biomasses. Lignin represents, however, a highly abundant natural aromatic feedstock with potentially very interesting applications in a sustainable economy scenario, especially considering the high primary production volumes and the inherently renewable nature of this resource [12]. Several studies are actually under way for the development of valuable lignin derivatives, such as raw chemicals [6], polymers [13,14,15,16,17,18], high-performance concrete admixtures [19,20], and hydrogels [21].

Different factors lead to the current marginal use of lignin as renewable feedstock, including its very complex and bulky macromolecular structure and its chemical recalcitrance in relation to the difficulties associated with the development of controlled depolymerization processes [12,22,23]. One of the main drawbacks in the full exploitation of the lignin value chain potentials is, however, its high variability. Unlike sugars, lignins are delivered as very complex mixtures and their structure and physico-chemical properties, especially in terms of composition, heterogeneity, and molecular size distribution, are strongly dependent on their natural origin (plant species and growing conditions) and on the specific process employed for their separation from the original biomass [1]. High-value lignin applications, such as the production of polymers and fine chemicals, typically require strict specifications of the starting material, particularly in terms of the chemical composition and molecular mass distribution. Variations in these characteristics due to different lignin origins and processing/extraction methods can cause significant alterations in the performance of the final product [24]. A critical dependence on lignin properties was found for several high-value applications [25,26,27,28], such as the production of polymers [16], antioxidants [29], aromatic compounds [30], the synthesis of vanillin [31], and the development of high-performance concrete water-reducing agents [32]. The availability of effective fractionation processes for obtaining lignins with tailored physico-chemical properties and a defined molecular weight distribution is therefore a key requirement to effectively cope with both the upstream material variability and the downstream application requirements.

Several innovative techniques are under study for the extraction of natural product from biomasses, including, among others, supercritical fluids, pulsed electric fields, and acoustic cavitation [33]. These are interesting approaches that are, however, still under development and not yet ready for a widespread implementation. In this work, the combined use of two well-known methods was instead addressed, namely solvent extraction and membrane ultrafiltration, in order to develop an enhanced lignin fractionation process using established technologies.

Solvent extraction is a widely adopted strategy to isolate technical lignin fractions with controlled properties [34,35,36,37]. This approach is typically implemented with the treatment of raw lignin using a sequence of different solvents with increasing solubilization capability in order to obtain selected fractions with defined characteristics. The main drawbacks of this method are the costs and the environmental issues related to the large amount of complex solvent systems involved in its implementation. Moreover, the characteristics of final fractions are strictly dependent on biomass–solvent interactions and variations in the lignin source can lead to the need of a whole process readjustment.

Membrane ultrafiltration is another interesting method for technical lignin fractionation [38,39]. Specific advantages are the ease of implementation, the low environmental impact, the high separation efficiency, and the consistency of results with different solutes and solvents. Membrane ultrafiltration is therefore a recognized approach for the development of a green separation process [40]. The main difficulty related to the application of this technology to lignin fractionation resides in the poor solubility of most technical lignins, with consequent membrane fouling and process performance degradation [41].

The combination of solvent extraction and membrane ultrafiltration appears as a promising strategy for the development of a viable lignin fractionation process. An application of this method was described for the delignification of non-woody lignocellulosic biomasses [42]. In previous works from our groups, an integrated approach was described that combines a first solubilization phase followed by membrane ultrafiltration in order to obtain a versatile cascade process for the fractionation of a technical lignin obtained from the soda process. This strategy was implemented with both aqueous [43] and organic [44] solvents. In the first case (hereafter referred to as the aqueous process), a solubilization in water/ethanol (60/40 *v/v*) mixture was performed, followed by a microfiltration step in order to obtain a clear lignin solution. In the second one (hereafter referred to as the organic process), the solubilization was carried out by Soxhlet extraction with the selected solvent. Both approaches implemented a two-section cascade membrane ultrafiltration downstream in order to obtain three different lignin fractions.

The combined strategy allows some drawbacks of each method to be overcome. The upstream solubilization phase allows the selection of the best conditions needed for each specific biomass. This ensures the delivery of a neat particle-free process fluid to the ultrafiltration system, with consequent mitigation of fouling-related problems. The downstream fine fractionation by membrane ultrafiltration allows in turn the use of a single solvent instead of a complex series needed for the implementation of the whole fractionation sequence with a multistage solvent extraction, providing great benefits in terms of decreased process costs, reduced complexity, and enhanced sustainability. Moreover, the integrated approach combines the versatility of the first solvent fractionation (inherent to the solubilization step) with the reliability and performance consistency of membrane ultrafiltration in the delivery of the final products.

In the present work, this approach was extended to two well-known technical lignins obtained from two distinct delignification processes (soda vs. kraft pulping) and originated from a non-woody (wheat straw/Sarkanda-grass) vs. a woody (softwood) biomass, respectively. In particular, a direct comparison between these two lignin feedstocks in terms of process yield and the chemical, physical, thermal, and structural characteristics of the resulting fractions was performed using both the aqueous and the organic processes. While previous studies focused on the investigation of single (either aqueous or organic) fractionation approaches, a comprehensive strategy was purposely developed here in order to maximize the control over the characteristics of the obtained lignins and to take advantage of the versatility of the proposed approach. Particularly, for both process implementations, the same type of membrane was adopted, allowing the use of a single ultrafiltration setup and ultimately optimizing the consistency of the two process outcomes. 

The approach presented in this work clearly demonstrates the versatility and the reliability of this integrated fractionation method, using two very different solvent systems and lignins of different origin and characteristics. Furthermore, the switchability between different solvents using the same ultrafiltration membrane systems enhances the potential of this fractionation method for a large variety of starting raw lignins, while the recyclability of the organic solvents used in the process in a close-circuit recirculation plant considerably reduces possible environmental concerns.

## 2. Results and Discussion

**Figure 1:**
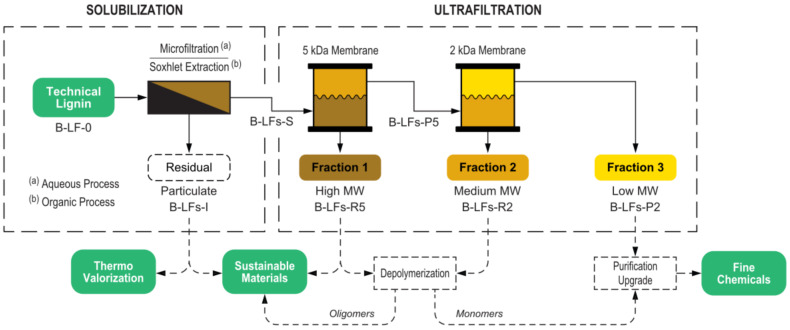
Overview of the overall fractionation process. Possible applications of obtained fractions and related downstream processes are indicated in the bottom line (dashed arrows).

This work demonstrates an integrated approach for lignin fractionation based on a cascade multistep process that combines a first solubilization step followed by a membrane ultrafiltration sequence. The process was implemented using both aqueous and organic solvents, and two different lignin feedstocks were investigated and thoroughly compared. In the aqueous process, the insoluble fraction was separated by microfiltration, while in the organic process, the solubilization was performed by Soxhlet extraction. Both processes included a cascade membrane ultrafiltration for the separation of fractions with tailored molecular weight distribution. The overall fractionation process is depicted in Figure 1.

**Figure 2:**
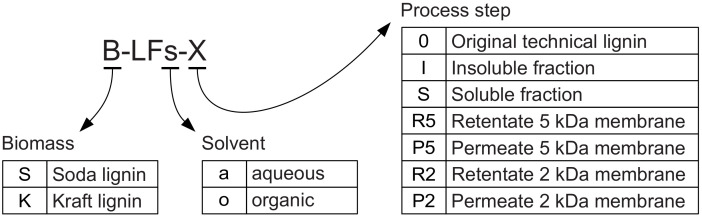
Labelling scheme for lignin fractions.

Because of the relevant number of different fractions handled, in Figure 1 and throughout the present work a labelling scheme for the working lignin fractions was adopted, allowing a quick identification of the related biomass, solvent, and process step (Figure 2).

A key design feature of this study was the implementation of the same ultrafiltration membrane setup for both the aqueous and the organic processes. This was obtained by the use of stabilized cellulose membranes, thanks to their very wide solvent compatibility that spans from water-based mixtures to a broad range of organic solvents. The possibility of using the same ultrafiltration membrane system with different process solvents is a very important result from a preparative perspective, allowing the selection of the most appropriate solubilization conditions for each specific biomass while maintaining high consistency in the delivered fractions. In addition, solvent switching costs are reduced, also as a result of the easy membrane cleaning (e.g., washing with 1 M NaOH solution at 40 °C and then conditioning with organic solvent for the aqueous to organic process switch). The simple process switching allows optimization of the fractionation conditions, mitigating the major drawbacks of each technology (i.e., membrane fouling and final solvent extraction in the case of the aqueous process, and the need of several different solvents in the case of the organic process).

The two processes were studied using two distinct model technical lignin systems, namely a wheat straw-Sarkanda grass lignin (Protobind 1000) obtained from the soda pulping process (S-LF-0) and a softwood lignin (Indulin AT) obtained from the kraft pulping process (K-LF-0). The soda lignin is a herbaceous sulphur-free lignin, whose chemical composition is close to that of natural lignin, given the relatively mild delignification conditions used to extract it. The choice of this commercially available lignin material for this study reflects the great impact of non-woody, agricultural, and crop-derived biomasses on the global biobased economy both in terms of economic turnaround and mass availability [13]. The kraft lignin is a byproduct of the sulphate cooking process and constitutes around 85% of the total world lignin production. During the kraft process, lignin is dissolved in severe conditions in an aqueous solution of sodium hydroxide and sodium sulphite and degraded into fragments. Kraft lignin contains a significant amount of sulphur as sulphonic groups, strongly affecting its solubility and ultimate chemical-physical characteristics.

### 2.1. Solubilization and Solvent Selection

The solubilization phase is the key step in order to allow the processing of different lignin sources. Particularly, the selection of the best solubilization conditions, governed in turn by the selection of the solvent process mixture, is of fundamental importance for the success of the fractionation methodology.

The aqueous process was performed by using a water-based solvent mixture that could be finely tuned by co-solvent selection and pH adjustment. In the present study, an ethanol/basic water mixture (60/40 *v/v*, adjusted to pH 10 with NaOH) was selected. The final pH after solubilization was around 8. This allowed a 75 and 95 g/L operating concentration for Soda (S-LFa-S) and Kraft lignin (K-LFa-S), respectively, to be reached. These conditions permitted working with a much higher lignin solution concentration with respect to the maximum value of 15 g/L obtained in a previous work for the same soda lignin [43] using pure water/ethanol (60/40 *v/v*). In the aqueous process, it was very important to implement a microfiltration step before the subsequent ultrafiltration in order to separate the insoluble fraction and avoid possible membrane clogging.

**Figure 3:**
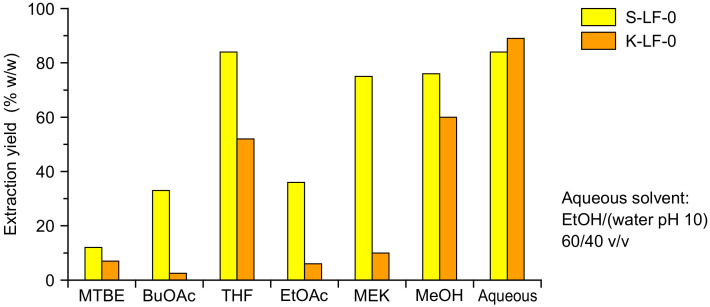
Extraction yields as the percent (*w/w*) of solubilized fraction vs. total fraction for S-LF-0 (yellow) and K-LF-0 (orange) for different organic solvents and for the (basic) aqueous system.

The organic process solubilization was performed with a Soxhlet extraction, which allowed the direct separation of the insoluble fraction and the recovery of a clean solute stream. The solvent selection is critical in order to obtain a viable process, with both biomass solubility and relevant solvent properties (e.g., easy evaporability because of high vapor pressure, low viscosity of the final solutions, recyclability, and uncritical handling) to be carefully considered. In order to determine the solubility characteristics of the two investigated technical lignins, a solubilization study was performed with six different organic solvents, namely *tert*-butyl methyl ether (MTBE), *n-*butyl acetate (BuOAc), tetrahydrofuran (THF), ethyl acetate (EtOAc), 2-butanone (MEK), and methanol (MeOH). The resulting extraction yields (as *w/w* percent of solubilized fraction vs. total fraction) are reported in Figure 3 (with aqueous solvent solubility as the comparison). 

As expected, marked differences were demonstrated for the solubility of the two technical lignins in the various organic solvents. The highest extraction yields (75~80%) were obtained in THF, MeOH, and MEK for S-LF-0, and in THF and MeOH (50~60%) for K-LF-0. Among these solvents, MEK presented several advantages in laboratory handling, including easy evaporation, good recyclability, uncritical handling, low toxicity, and low viscosity of the extracts (that significantly limited ultrafiltration membrane clogging issues). Because of these benefits, and considering also its extensive use at the industrial scale, MEK was selected in this study as the working organic solvent for both the soda and kraft lignin fractionation. This also led to a comparison of the process performances in both high solubilization conditions (with S-LF-0) and low solubilization conditions (with K-LF-0). The obtained operative biomass concentrations in the extracted MEK solutions were 95 g/L for soda (S-LFo-S) and 15 g/L for kraft (K-LFo-S) lignin.

### 2.2. Membrane Ultrafiltration

The membrane ultrafiltration phase was pivotal for the quality and consistency of the lignin fractions delivered by the process. As the key design requirement was the implementation of the same membrane set for both aqueous and organic processes, two modified cellulose ultrafiltration membranes (with a nominal cut-off at 5 and 2 kDa) were selected for their wide solvent and pH compatibility. This selection allowed the limited solvent compatibility of the polyether sulfone ultrafiltration membranes used in a previous work to be overcome [43]: These membranes in fact were found to work quite well in aqueous solution but suffered from limited compatibility with organic solvents. In this study, process parameters, such as the flow rate, pressure, temperature, and recirculation cycle, were thoroughly optimized according to the requirement of each solvent. As will be discussed in the following sections, the limited dependence of the molecular weight distribution of delivered fractions on the solvent used confirmed a good consistency of the biomass fractionation products.

### 2.3. Lignin Fractionation

According to the results discussed in Section 2.1 and reported in Figure 3, the aqueous process was designed for a starting solution of lignin (both kraft and soda) in ethanol/water (60/40) in basic conditions. Preparation of the lignin starting solution was optimized by adding sodium hydroxide to facilitate the solubilization process. The final pH of the solution was around 8. The sample recovery from the aqueous process fractions included four main steps: Evaporation under vacuum of the ethanol present in the solutions; acidification of the aqueous solutions with hydrochloric acid (or sulfuric acid) at a pH close to 1; extraction in organic solvent, such as ethyl acetate; and evaporation of the organic phase and recovery of the solid fractions. On the other hand, the fractions coming from the organic process, after recovery, were evaporated under reduced pressure without further processing.

The best suitable concentrations for S-LF-0 and K-LF-0 in the aqueous process were found to be around 75 and 95 g/L, respectively, whereas the best concentrations in the organic process were 95 and 15 g/L due to the great chemical differences between the two lignins. 

Aliquots of the permeates and retentates obtained from S-LF-0 and K-LF-0 for the two fractionation processes (aqueous and organic) were withdrawn after successive steps and fully characterized in terms of the chemical composition, molecular weight, and physical properties. It should be pointed out that the determination and the possible variations in the molecular weight and the composition of the lignin fractions are fundamental for their successful valorization as precursors of chemicals and polymeric materials. Moreover, the quantification and characterization of the functional groups present in the samples constitute essential steps for the effective assessment of a lignin valorization setup. Therefore, one of the goals of this work was the extraction and the full characterization of each fraction in order to recover homogeneous fractions with specific properties.

### 2.4. Gel Permeation Chromatography (GPC)

GPC analyses were performed on all fractions in order to evaluate the effectiveness of the aqueous and organic processes on the molecular weights and molecular weight distributions of the technical lignins considered in this work.

The characteristic values of the number-average molecular weight (Mn), weight-average molecular weight (Mw), and polydispersity index (*Đ*) are listed in Table 1 for S-LF-0 and Table 2 for K-LF-0. Analyses were performed using mono-dispersed polystyrene standards as the reference (see the materials and methods section for details).

Both in the aqueous and in the organic process, the soda and kraft lignins exhibited a similarly noticeable decrease in their molecular weight after permeation through membranes of increasingly finer mesh. In particular, the filtration through a 2-kDa cut-off membrane was shown in all cases to provide lignin fractions with Mn in the range of ~500 g/mol, both for soda and kraft lignin. Such a reduction in the molecular weight was accompanied by a significant narrowing of the molecular weight distribution, as evidenced by the lower value of *Đ* obtained in the lighter fractions (*Đ* = 1.2 for S-LFa-P2 and S-LFo-P2, *Đ* = 1.5 for K-LFa-P2 and K-LFo-P2) with respect to the parent lignin material (*Đ* = 3.3 and 2.7 for S-LF-0 and K-LF-0, respectively). It is also evident how retentate fractions in general exhibited higher molecular weights and larger *Đ* values when compared with permeate fractions, likely due to a broader distribution of longer lignin macromolecules in the former. These trends are in line with previously reported results on both kraft and soda lignin fractionation processes selectively undertaken in organic solvent or the aqueous phase [39,43,44,45,46]. Interestingly, fraction K-LFa-R5 was found to be poorly soluble in the solvent used as eluting medium for the GPC analysis (THF), leaving a large amount of solid residue as precipitate even after acetylation. This result suggests a significant enrichment in high molecular weight chains in the retentate material of kraft lignin after the first aqueous-based ultrafiltration step (5 kDa cut-off). 

**Figure 4:**
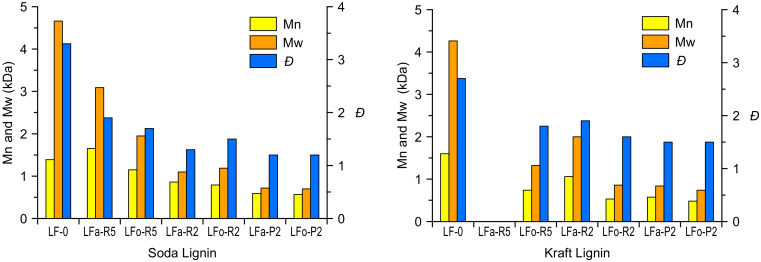
Comparison of the results of the aqueous and organic processes for S-LF-0 and K-LF-0 fractionation as Mn, Mw, and *Đ* by GPC analysis of resulting delivered fractions.

Finally, it is worth noting that the same trends on the obtained molecular weights (decreasing) and molecular weight distributions (narrower) for lignin fractions recovered after filtration through increasingly finer membranes were observed in both aqueous and organic processes (Figure 4). In particular, these trends were found to be comparable for soda and kraft lignins. This evidence highlights the key role played by the ultrafiltration process in providing consistent and reproducible fractions of narrowly controlled molecular characteristics. In addition, it suggests that the performance of the ultrafiltration membranes is not significantly affected by the solvent used, indicating a factual independence between the extraction operation (controlled by the solvent) and the downstream fractionation (controlled by the ultrafiltration membranes). This aspect is a very relevant feature for an industrial scale-up perspective because it represents a clear advantage in terms of process versatility and straightforward optimization when tackling different types of biomass as input feedstock.

### 2.5. Gel Filtration Chromatography (GFC)

**Figure 5:**
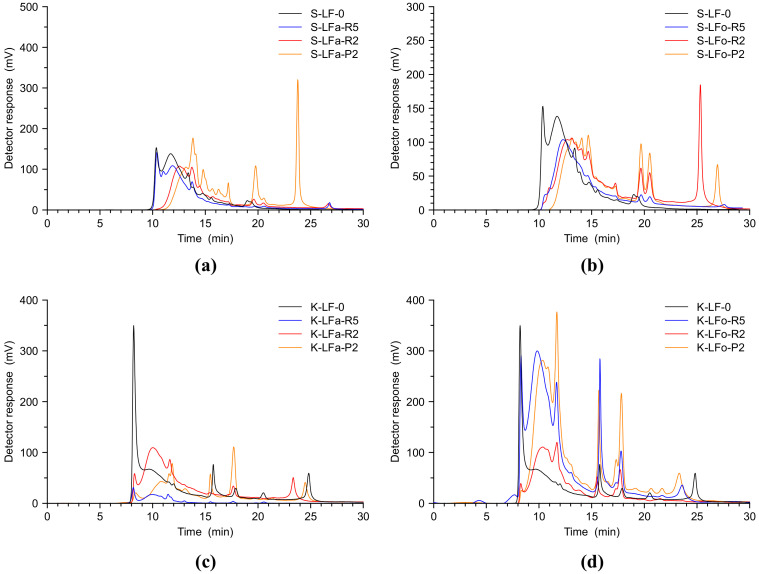
GFC chromatograms of selected soda (**a**,**b**) and kraft (**c**,**d**) lignin fractions recovered from the aqueous process (**a**,**c**) and the organic process (**b**,**d**).

GFC analyses are reported in Figure 5 below, showing the performance of the two fractionation processes for S-LF-0 and K-LF-0, respectively. A new procedure using a basic water solution as eluent was developed in this work. Moving through the fractionation processes, the GFC analysis showed very well the shift of the peaks towards lower retention times, proving the effectiveness of the proposed approach in a straightforward manner. As it is clear in Figure 5, moving from the pristine lignin (in black), to the retentate at 5 kDa (in blue) and more clearly to the chromatogram of the fractions exiting the membrane with the finest cut-off (permeate B-LFo/a-P2, 2 kDa membranes, yellow lines), in all cases the enrichment in small molecules represented by the peaks at a higher retention time was striking. This analysis demonstrated that the two fractionation processes were very effective even when applied to two different biomasses.

### 2.6. Gas-Chromatography/Mass Spectrometry (GC/MS)

The identification and quantification of low molecular weight compounds present in each fraction was performed by using GC/MS. This analytical tool allowed the analysis of the distribution of the small compounds present in each fraction, divided into three main classes—namely, benzaldehydes and acetophenone derivatives (ArCHO, ArCOR), benzoic and coumaric acids (ArCOOH, ArCHCHCOOH), and aliphatic long-chain carboxylic acids (RCOOH). 

The analyzed samples were prepared with a small-scale chromatography on silica gel in order to eliminate all the polymeric/oligomeric fractions and to recover only the suitable fraction for GC/MS analysis (% of mass recovered after chromatography is reported in Table 3 and Table 4). The distribution trends confirmed clearly the efficacy of the fractionation procedures for the two considered lignins, as the percentages of total monomers in each sample increase when proceeding through the two processes. As reported in Table 3 for S-LF-0 fractionation, the percentage of monomers recovered through the aqueous process, which were analyzed in GC/MS, moved from 8% (*w/w*) of monomers in the higher molecular weight fraction to a maximum of 81% (*w/w*) in the lower molecular weight one whereas in the organic process the percentage of monomers shifted from 7% to 63%.

For K-LF-0 fractionation, the results of the GC/MS analysis are reported in Table 4. Additionally, in this case, the efficacy of the fractionation procedure, expressed as percentages of total monomers in each sample, increased when proceeding through the processes: K-LFa-R5 and K-LFo-R5, constituting the higher molecular weight retentates, contain only 21% of small molecules, which could be inferred from GC-MS; on the contrary, K-LFa-P2 and K-LFo-P2, constituting the fractions permeated from the finest membrane (2 kDa cutoff), were considerably enriched in small molecules (around 70%).

**Figure 6:**
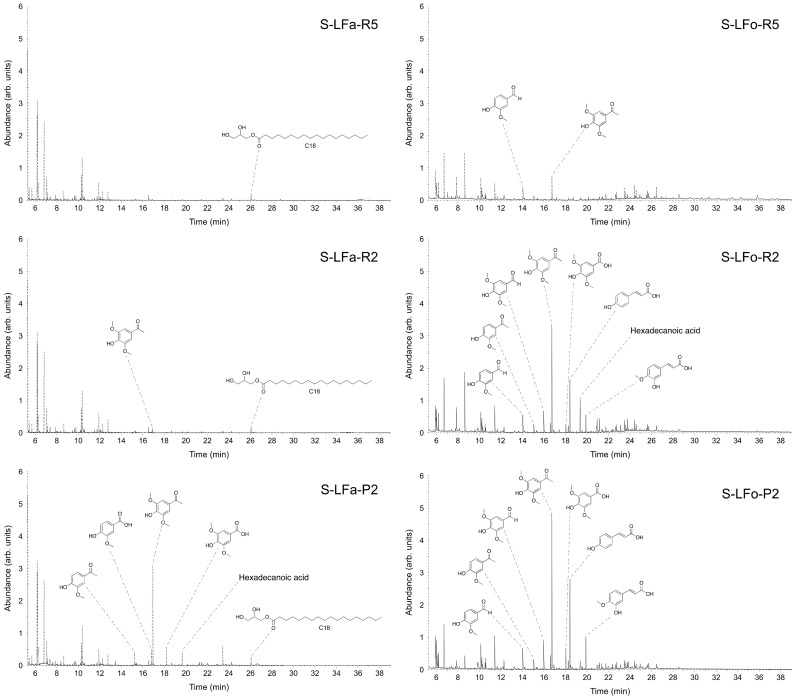
GC/MS total ion chromatograms of delivered fractions from aqueous and organic processes using S-LFa/o-0. Selected component identification by MS library matching is reported for each fraction.

**Figure 7:**
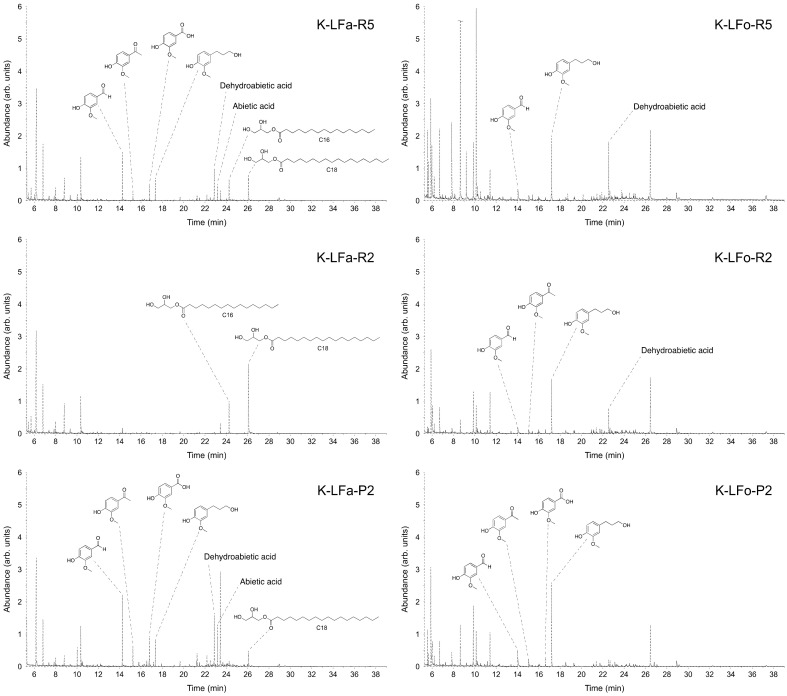
GC/MS total ion chromatograms of delivered fractions from aqueous and organic processes using K-LFa/o-0. Selected component identification by MS library matching is reported for each fraction.

The chromatogram profiles of the different fractions and the structures of the most abundant identified compounds with their relative abundance are reported in Table 3 and Figure 6 for S-LF-0 and Table 4 and Figure 7 for K-LF-0. As discussed above (Table 3 and Table 4), the enrichment in smaller molecular weight products clearly appeared when moving throughout the fractionation processes for S-LF-0 from fractions S-LFa/o-R5 to S-LFa/o-P2 (as shown in Figure 6), whereas for K-LF-0 the number of peaks in the chromatograms becomes much more relevant in K-LFa/o-P2 (in Figure 7). The peaks from 7 to 12 min are mainly due to the silylating agent used for the derivatization of the analyzed samples. The peak at 6 min is attributed to benzaldehyde, which was used as the internal standard.

### 2.7. Determination of the Hydroxylation Levels in the Recovered Fractions

The determination of hydroxylation levels in lignins constitutes an essential step for the effective valorization of the fractions obtained from the fractionation processes. The classical methods are the Folin–Ciocalteu titration method (FC), which allows the quantification of phenolic groups, and ^31^P-NMR analysis, which leads to a quantitative determination of aliphatic and aromatic hydroxyl groups as well as of carboxyl moieties.

#### 2.7.1. Phenolic Hydroxyl Group Determination

The FC assay was carried out to determine the total phenolic content in the original lignin and all process fractions. It is based on the reaction of phenolic hydroxyl groups with a specific redox reagent (FC reagent), which leads to the formation of a blue chromophore, which is, however, sensitive and unstable in strong bases. Therefore, based on an analytical protocol previously demonstrated by our group [41], DMSO was used as solvent for the samples in order to obtain their complete solubilization in neutral conditions. 

The phenolic content results are reported in Table 5 and Table 6 as vanillin equivalents (mmol/g of dry lignin). In the case of soda lignin, S-LFa/o-R2 and S-LFa/o-P2 appeared to be the fractions with a higher content in phenolic hydroxyl groups, in agreement with the trends observed with ^31^P-NMR, as will be discussed later in the text.

If we compare the two starting lignins, K-LF-0 appears to contain an increased amount of phenolic hydroxyl groups compared to S-LF-0, probably due to the extensive cleavage of β-aryl group bonds during the cooking process. In S-LF-0 fractionation, a very high enrichment in phenolic groups appears in the fractions R2 and P2 as reported in Table 5, in excellent agreement with the GC/MS analysis reported in Figure 6. For K-LF-0 fractionation, as reported in Table 6, the highest value of phenolic content appears to be associated with K-LFa-P2 for the aqueous process, whereas in the organic one, the R5 fraction appears to be enriched in phenolic groups. 

#### 2.7.2. Total Hydroxyl Groups’ Quantification with ^31^P-NMR

**Figure 8:**
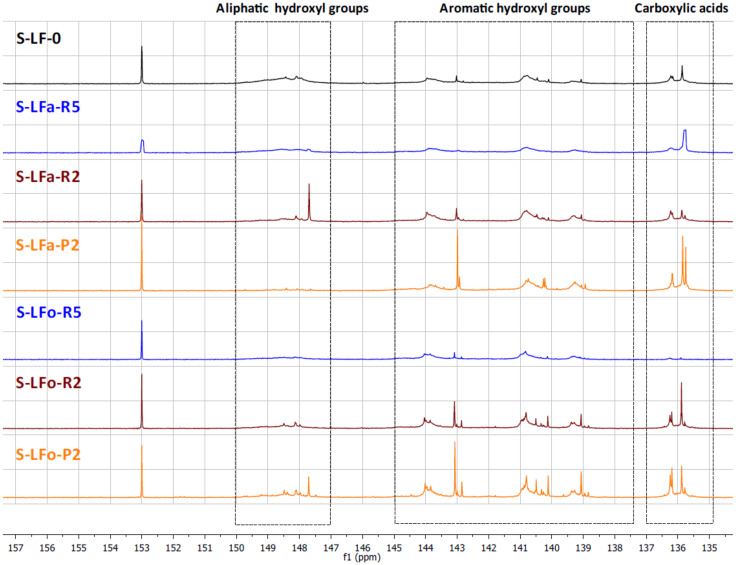
^31^P-NMR spectra of lignin fractions recovered from the aqueous and the organic process applied on S-LF-0.

**Figure 9:**
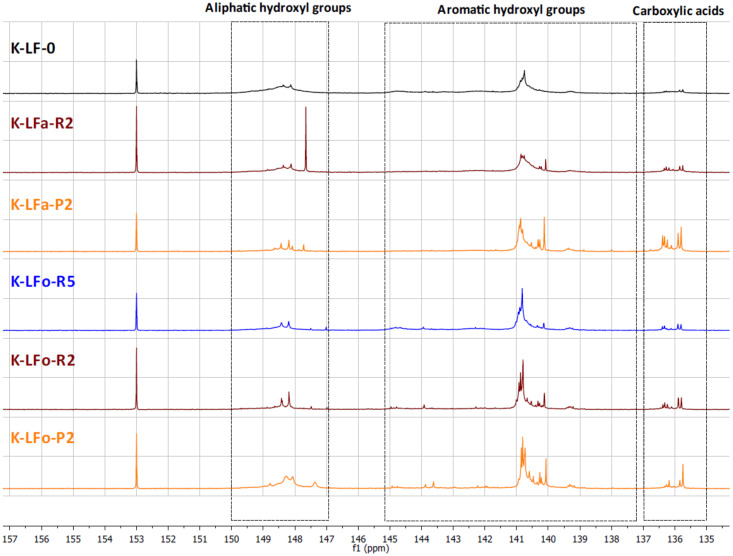
^31^P-NMR spectra of lignin fractions recovered from the aqueous and the organic process applied on K-LF-0.

The different types of hydroxyl groups present in the starting lignins and fractionated samples were deeply investigated by ^31^P-NMR. The ^31^P-NMR spectra of the phosphitylated lignin fractions are shown with chemical shift assignments in Figure 8 and Figure 9, where the parent materials S-LF-0 and K-LF-0 are also shown for easy comparison. In detail, the following spectral regions were integrated to acquire the information about the chemical nature of the different hydroxyl groups: 150–147 ppm for the signals associated with aliphatic hydroxyl groups, and 145–138 ppm for the signals associated with aromatic hydroxyl groups. The signals of carboxylic acids groups were centered at 136 ppm. The peak integration in the three main portions of the spectrum led to the quantification of the total hydroxyl groups expressed in mmol of functional groups per gram of dry compound, as reported in Table 7 and Table 8 for S-LF-0 and K-LF-0, respectively.

Both FC assay and ^31^P-NMR data indicated a slight increase in phenolic hydroxyl groups in the lower molecular weight fractions B-LFa/o-R2 and B-LFa/o-P2. A similar trend was also reported from ^31^P-NMR analysis on aliphatic hydroxyl and carboxyl groups, in agreement with recent works on analogous technical lignins [43,44,45]. These results have great interest in terms of further selection of an appropriate fraction for a specific application, where the relative abundance of the functional groups is essential for the further processing.

### 2.8. Fourier-Transform Infrared Spectroscopy (FTIR)

**Figure 10:**
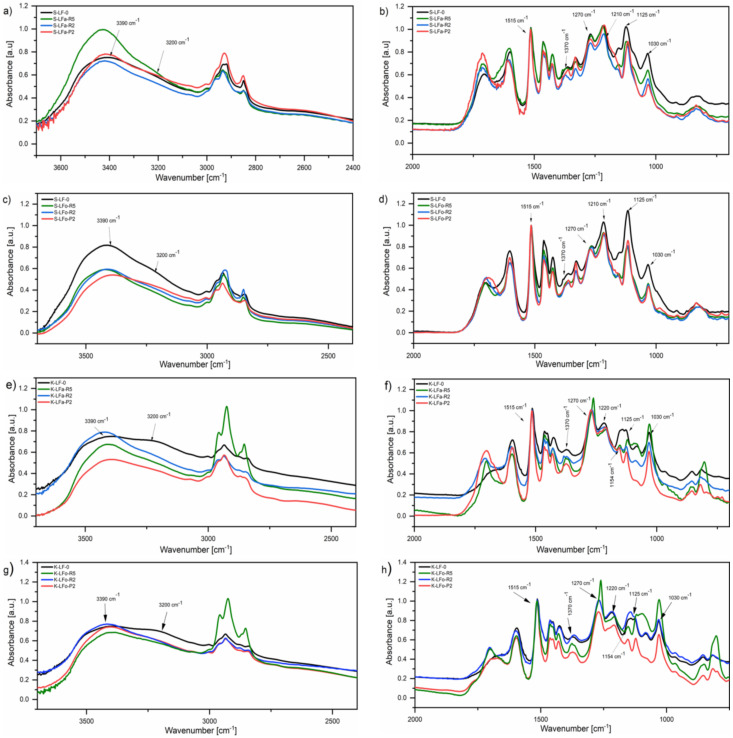
FT-IR spectra of soda (**a**,**b**,**c**,**d**) and kraft (**e**,**f**,**g**,**h**) lignin fractions recovered from the aqueous process (**a**,**b**,**e**,**f**) and the organic process (**c**,**d**,**g**,**h**). Different regions of the infrared spectrum are reported for clarity.

FTIR spectroscopy was employed to analyze the chemical characteristics of the lignin fractions recovered after solvent extraction and subsequent membrane-assisted ultrafiltration. FTIR spectra of both the soda and kraft lignin fraction systems are presented in Figure 10, along with the FTIR spectrum of each parent lignin also shown for comparisons. All fractions presented a broad absorption band in the 3600–3100 cm^−1^ range that could be attributed to stretching vibrations of hydrogen-bonded phenolic and aliphatic O−H groups present in lignin, with a peak at 3390 cm^−1^ and a shoulder at 3200 cm^−1^, respectively [47]. Signals observed in the 3050−2800 cm^−1^ region were attributed to the symmetrical and asymmetrical CH stretching of the methyl and methylene groups. At around 1700 cm^−1^, a clear peak was observed in all spectra with the exception of K-LF-0, where more of a shoulder was observed, attributable to the stretching vibration of C-O bonds in conjugated aldehydes and carboxylic acids. In particular, it was observed that after solvent extraction (in both kraft and soda lignin streams), an increase in this signal was registered compared with the pristine samples (especially in low molecular weight fractions S-LFa/o-P2 and K-LFa/o-P2), likely indicating a higher concentration of carbonyl and carboxylic groups. The presence of the intense peak at 1515 cm^−1^ was associated to the pure aromatic skeletal vibrations in lignin. In the 1400−1000 cm^−1^ spectral region, signals of variable intensity were observed, which could be attributed as follows: Bending vibrations of phenolic O−H and aliphatic C−H in methyl groups (1370 cm^−1^); C−O, C−C, and C−O stretching vibrations (1270 and 1210 cm^−1^); C−H in plane deformations (1125 cm^−1^); and C−O deformations in primary (1030 cm^−1^) alcohols. 

In the kraft fractions, both in aqueous and organic solvent, a clear peak at 1154 cm^−1^ was observed, which could be assigned to C-O deformations in conjugated ester groups present in guaiacyl (G), syringyl (S), and *p*-hydroxyphenyl (H) groups. This signal indicated a higher abundance of such moieties, not present in soda fractions.

### 2.9. Differential Scanning Calorimetry (DSC)

**Figure 11:**
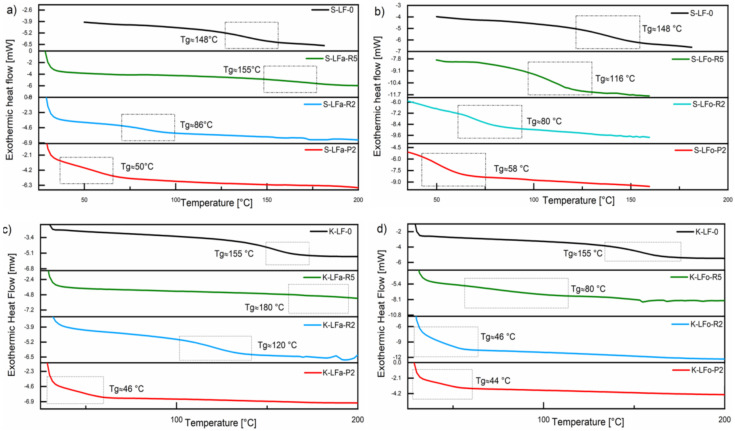
DSC traces of soda (**a**,**b**) and kraft (**c**,**d**) lignin fractions recovered from the aqueous process (**a**,**c**) and the organic process (**b**,**d**).

The thermal transitions in all lignin fractions, in particular the glass transition temperature (Tg), were evaluated by means of DSC analysis and compared with the pristine samples S-LF-0 and K-LF-0. DSC traces of both soda and kraft lignin fraction systems are presented in Figure 11. As it can be seen in the plots, in the case of organic solvent fractionation, the solvent extraction step led to a decrease in Tg of all the recovered materials, with samples S-LFo-P2 (Tg = 58 °C) and K-LFo-P2 (Tg = 44 °C) showing the lowest values, in accordance with the trends observed on molecular weights in the GPC results and in line with what was recently reported on analogous systems [43,44]. In the case of aqueous fractionation, a similar trend was observed on S-LFa-P2 and K-LFa-P2, with comparable values of Tg (Tg = 50 °C and 46 °C for S-LFa-P2 and K-LFa-P2, respectively). Sample S-LFa-R5 showed a slightly higher Tg than the parent sample S-LF-0, likely due to the slightly higher value of Mn observed for this fraction in GPC measurements. Similarly, the Tg of sample K-LFa-R5 was not easily detectable from the DSC trace but was higher (Tg = 180 °C) than the corresponding parent material K-LF-0 (Tg = 155 °C), in line with the high molecular weight inferred for this fraction from GPC analysis (Table 2).

The similar trends observed on the resulting Tg of the two different lignin systems (soda vs. kraft) upon fractionation in both aqueous and organic phase further demonstrated the versatility of the proposed process, which did not appear to be significantly affected by the solvent used for the extraction operation. Moreover, they provided additional proof of the ability of this approach to deliver consistent and reproducible fractions of well-controlled and predictable thermal characteristics irrespective of the biomass origin, with this aspect being particularly useful for potential use in the development of lignin-based macromolecular materials.

## 3. Materials and Methods

### 3.1. Materials

All chemicals and analytical grade solvents, such as tetrahydrofuran (THF), methanol (MeOH), ethyl acetate (EtOAc), n-butyl acetate (BuOAc), and *tert*-butyl methyl ether (MTBE), were purchased from Sigma-Aldrich. 2-Butanone (methyl ethyl ketone, MEK) was provided by BCD Chemie GmbH (Hamburg, Germany). Lignin S-LF-0 (Protobind 1000, a mixed wheat straw/Sarkanda grass lignin from soda pulping of non-woody biomass) was provided by Tanovis (Alpnach, Switzerland). Lignin K-LF-0 (softwood Kraft lignin, Indulin AT) was provided by Meadwestvaco (Charleston, SC, USA). Lignins were used as received. All solutions were prepared in Milli-Q water (Elix Millipore Purification System, Molsheim, France). All analyses were carried out at least in duplicate unless otherwise stated.

### 3.2. Lignin Solubility in Organic Solvents

Lignin solubilities in organic solvent were determined by treating 10 g of starting lignin with 100 mL of the different solvents stirring at 400 rpm. Each test was carried out overnight at room temperature. The suspensions were then filtered, and the solvents were evaporated at reduced pressure and the final residues were dried until a constant weight was achieved prior to quantification. 

### 3.3. Fractionation via Aqueous Process

The aqueous process fractionation comprised a microfiltration step followed by a two-membrane cascade ultrafiltration (shown in Figure 1 as part of an idealized lignin valorization scheme) and a final fraction recovery step, as described below (see also the discussion section).

#### 3.3.1. Microfiltration

The lignin solution in ethanol/ water was prepared by dissolution of 150 g of lignin per L of a mixture of ethanol and NaOH aqueous solution at pH 10. The insoluble material was eliminated by a microfiltration on a 0.7-μm fiberglass filter under vacuum.

#### 3.3.2. Membrane-Assisted Fractionation

Lignin fractionation was performed by means of an ultrafiltration apparatus (Sartoflow Advanced filtration module purchased from Sartorius Stedim, Aubagne, France) equipped with flowmeters and pressure sensors to control the permeate flow, trans-membrane pressure, and filtration time. The membranes (Hydrosart membranes, Sartorius Stedim) in stabilized cellulose had nominal molecular weight cutoffs of 5 and 2 kDa and a filtration area of 0.1 m^2^ each. The membrane regeneration and storage were performed at 40 °C using 1 M and 0.1 M NaOH solution, respectively.

These membranes were compatible with a high range of different solvents, allowing to switch easily from an aqueous solvent to an organic one. The procedure required the cleaning of the membrane with a solution of 1M NaOH at 40 °C, and then the washing and conditioning of the membrane in the appropriate solvents for the successive fractionation sequence. The membranes could be also stored without any problems in 0.1 M NaOH.

#### 3.3.3. Fraction Extraction Procedure

The recovered fractions from the aqueous process were evaporated under reduced pressure to remove ethanol. The aqueous solutions were acidified to pH close to 1 with 37% HCl, and extracted three times with ethyl acetate. The combined organic phases were dried over sodium sulfate and evaporated under reduced pressure. The final solid residues were dried until a constant weight was achieved prior to analysis and quantification. 

### 3.4. Fractionation via Organic Process

The organic process fractionation comprised a Soxhlet extraction followed by a two-membrane cascade ultrafiltration (shown in Figure 1 as part of an idealized lignin valorization scheme) and a final fraction recovery step, as described below (see also the results and discussion section).

#### 3.4.1. Soxhlet Extraction

Soxhlet extractions were performed using a standard glass apparatus (Buchi extraction system B-811, Villebon sur Yvette, France), which allowed 4 extractions to be achieved at the same time. Each extraction unit was composed of a 150-mL working volume bottom solvent flask, a 330-mL-capacity thimble holder and a water-cooled condenser. For each extraction process, 150 mL of MEK were placed in the solvent flask and about 15 g of lignin were inserted inside a 41-mm diameter and 123-mm height cellulose paper thimble (FiltraTECH, Saran, France). The solvent reflux was kept for 8 h, adjusting the heating power in order to have 4 extraction cycles/h.

#### 3.4.2. Membrane Ultrafiltration

Lignin fractionation was performed in MEK directly on the Soxhlet extraction solution by means of the same apparatus described in Section 3.3.2 with the membranes conditioned and used only in MEK.

#### 3.4.3. Fraction Recovery Procedure

The recovered fractions from the organic process were evaporated under reduced pressure without further processing. The final solid residues were dried until a constant weight was achieved prior to analysis and quantification. 

### 3.5. Gel Permeation Chromatography (GPC)

A Waters 510 HPLC system equipped with a refractive index detector was used for GPC analyses. Tetrahydrofuran (THF) was used as eluent. The analyzed lignin sample (volume 200 μL, concentration 1 mg/mL in THF) was injected into a system of three columns connected in series (Ultrastyragel HR, Waters–dimensions 7.8 mm × 300 mm) and the analysis was performed at 30 °C at a flow rate of 0.5 mL/min. The GPC system was calibrated against polystyrene standards in the 10^2^–10^4^ g/mol molecular weight range. To allow complete solubility in the THF eluent, before the analysis, the parent lignin and the fractions were acetylated following a standard literature procedure. The estimation of the number-average and weight-average molecular weights of the obtained lignin fractions was performed excluding the signals related to the solvent (THF) and the solvent stabilizer (butylated hydroxytoluene), visible at long elution times (>29.5 min).

### 3.6. Gel Filtration Chromatography (GFC)

GFC analyses were performed using a Merck-Hitachi L4000 apparatus (Tokyo, Japan) equipped with a UV detector set at λ of 254 nm. A hydroxylated acrylic polymer GFC column (Polysep-GFC-P 2000, 300 × 7.8 mm, Phenomenex, Aschaffenburg, Germany) was used with a water-based eluent (sodium borate pH 10 buffer 10 mM plus 300 mM NaCl) at 0.5 mL/min flow rate. 

The samples (5 mg) were completely dissolved in a small amount of NaOH. The pH of the solution was adjusted to pH 10 with HCl 6 M, and diluted to the final concentration of 2 mg/mL with sodium borate buffer 10 mM pH 10. The sample was then centrifugated, filtered, and analyzed.

### 3.7. Gas-Chromatography/Mass Spectrometry (GC/MS)

The GC/MS apparatus used was an Agilent GC System 7890A, with an inert MSD with Triple-Axis Detector 7975C (Cernusco sul Naviglio, Italy). The separation was performed on a DB-5MS column (30 m × 250 μm × 0.25 μm, Phenomenex) with a helium flow rate of 1.18 mL/min, a temperature program of 50 °C (1 min) to 280 °C at 10 °C/min, 280 °C at 15 min (total run time 39 min, temperature of the injector 250 °C, injection volume 1.00 μL, injection mode split, split ratio 5:1). A solvent delay of 4 min was selected. The samples were prepared by derivatization and dissolved in methanol or acetone in a concentration around 0.5-1 mg/mL as previously described [44]. Compound identification was performed by means of an NIST 2008 mass spectral library search.

### 3.8. Folin–Ciocalteu Assay (FC Assay)

Total phenolic contents of the different fractions were determined by the classical Folin–Ciocalteu method with some modifications in the sample preparation step. The samples were prepared by dissolving lignin in dimethylsulfoxide (DMSO) with a final concentration of 1 mg/mL. DMSO was chosen because, being completely miscible in water, it allowed a complete lignin solubilization and did not interfere with the FC assay. 

For each determination, 5 µL of the working solution (or the standard solution) were then mixed with 120 μL of deionized water, 125 μL of FC reagent (Sigma 47641), and kept for 6 min at r.t. after 30 s of vortex stirring. Then, after the addition of 1.25 mL of 5% sodium carbonate and mixing, the vial was incubated on a thermoshaker at 40 °C for 30 min. The reaction mixture absorbance was measured using a UV/Vis spectrophotometer (Jasco V-560) equipped with a temperature-controlled cuvette holder and a thermostatic water bath (Haake K10, Karlsruhe, Germany). All spectrophotometric measurements were carried out at 760 nm, 25 °C, using a 1-cm optical path cuvette. Vanillin was chosen as the reference standard. The calibration curve was constructed with nine different vanillin solutions in DMSO with the concentration in the range 0–800 μg/mL. Each FC assay determination was carried out in triplicate.

### 3.9. ^31^P-NMR Analysis

^31^P-NMR spectroscopic analyses were recorded on a Bruker Instrument AVANCE400 spectrometer (Milano, Italy). Acquisition and data treatment were performed with Bruker TopSpin 3.2 software (Milano, Italy). The spectra were collected at 29 °C with a 4-s acquisition time, 5-s relaxation delay, and 256 scans. Prior to analysis, samples were dried for 24 h under vacuum and then derivatized according to the following procedure. 

A lignin sample (40 mg) was completely dissolved in 300 μL of *N*,*N*-dimethylformamide. To this solution, the following components were added: 200 μL of dry pyridine, 100 μL of solution of internal standard (10 mg of Endo-*N*-hydroxy-5-norbornene-2,3-dicarboximide (Sigma 226378) dissolved in 0.5 mL of a mixture of pyridine and CDCl_3_ 1.6:1 *v/v*), 50 μL of solution of relaxation agent (5.7 mg of chromium (III) acetylacetonate (Sigma 574082) dissolved in 0.5 mL of a mixture of pyridine and CDCl_3_ 1.6:1 *v/v*), 100 μL of 2-chloro-4,4,5,5-tetramethyl-1,3,2-dioxaphospholane (Sigma 447536), and at the end 200 μL CDCl_3_. The solution was centrifuged if necessary. All chemical shifts reported were related to the reaction product of the phosphorylating agent with water, which gave a signal at 132.2 ppm.

### 3.10. Fourier-Transform Infrared Spectroscopy (FT-IR)

FT-IR spectra of all lignin fractions were recorded in transmission mode on lignin-containing KBr pellets (amount of lignin was approximately 5 mg). The analysis was performed by means of a Nicolet 760-FTIR spectrophotometer (Thermo Fisher Scientific, Rodano, Italy) at room temperature in air in the 4000−700 cm^−1^ wavenumber range with 64 accumulated scans and a resolution of 2 cm^−1^.

### 3.11. Differential Scanning Calorimetry (DSC)

DSC was performed on solid-state samples (about 10–15 mg) by means of a Mettler-Toledo DSC/823e instrument (Milano, Italy) at a scan rate of 20 °C/min under nitrogen flux.

## 4. Conclusions

Lignin constitutes a byproduct accumulated worldwide in significant quantities in the pulp and paper industry, despite possessing a great potential for generating novel biobased platform chemicals and polymeric materials alternative to those currently derived from petrochemical routes. One of the most important tasks when targeting the establishment of any lignin valorisation process is the ability to set up robust and reliable fractionation processes enabling the recovery of lignin streams with controllable and predictable composition and properties. Indeed, the great variability in its chemical composition, structural characteristics, molecular weight, and reactivity, intimately linked to the vast geographical and seasonal distribution of lignocellulosic biomass feedstocks, makes lignin processing and valorization particularly challenging. 

In an attempt to address this issue, in this work, a straightforward fractionation method was demonstrated combining solvent extraction and membrane-assisted ultrafiltration. This approach was applied to two commercially available technical lignins with different origins (non-woody vs. woody biomass) obtained from two distinct delignification routes (soda vs. kraft pulping), starting from an extraction process using two different solvent (aqueous vs. organic) systems. Based on this strategy, a comprehensive method was demonstrated for the tunable and predictive control over the characteristics of the recovered lignin fractions. In particular, the key role played by the ultrafiltration process in providing consistent and reproducible fractions of narrowly controlled chemical, structural, molecular, and thermal characteristics was shown. In addition, the performance of the ultrafiltration membranes was found to be relatively unaffected by the solvent used (either aqueous or organic), indicating a relative independence between the extraction operation (controlled by the solvent) and the downstream fractionation (controlled by the ultrafiltration membranes). This aspect is a very relevant feature from an industrial scale-up perspective, because it represents a clear advantage in terms of process tunability and optimization when tackling different types of biomass as input feedstock. 

This versatile, robust, and easy to implement fractionation approach paves the path for the delivery of consistent and reproducible lignin fractions of well-controlled and predictable properties irrespective of the biomass origin, with this aspect being of particular interest in view of their potential use in the development of lignin-based macromolecular materials.

## Figures and Tables

**Table 1:** GPC analysis (molecular weights Mn and Mw, polydispersity index *Đ* of all examined soluble lignin fractions from S-LF-0 obtained with the two fractionation methods).

Sample ^a^	% of Starting Material (*w/w*)	Mn (Da)	Mw (Da)	*Đ*
S-LF-0	-	1390	4660	3.3
S-LFa-R5	49.3	1650	3090	1.9
S-LFa-R2	7.6	860	1100	1.3
S-LFa-P2	6.0	590	720	1.2
S-LFo-R5	66.3	1150	1950	1.7
S-LFo-R2	7.0	790	1190	1.5
S-LFo-P2	1.6	570	700	1.2

^a^ Samples were eluted after acetylation. The reported values for molecular weights are relative to polystyrene standards.

**Table 2:** GPC analysis (molecular weights Mn and Mw, polydispersity index *Đ* of all delivered fractions of aqueous and organic processes fractionation of K-LF-0.

Sample ^a^	% of Starting Material (*w/w*)	Mn (Da)	Mw (Da)	*Đ*
K-LF-0	-	1600	4260	2.7
K-LFa-R5 ^b^	72	n.a.	n.a.	n.a.
K-LFa-R2	4.8	1060	2000	1.9
K-LFa-P2	2.5	575	840	1.5
K-LFo-R5	7.9	740	1320	1.8
K-LFo-R2	1.7	530	860	1.6
K-LFo-P2	1.1	480	740	1.5

^a^ Samples were eluted after acetylation. The reported values for molecular weights are relative to polystyrene standards. ^b^ This fraction was found to be poorly soluble in the eluting solvent (THF) even after acetylation, preventing the possibility of performing GPC analysis.

**Table 3:** Summary of the GC/MS results of fractions coming from aqueous and organic processes applied on S-LF-0 illustrating the estimated amount of volatile compounds, divided in three main groups. Data reported as the total mass of compounds/mass of initial fraction (%). Values were rounded up to the nearest two significant figures with an estimated relative error of ± 0.01.

Compounds	S-LFa-R5	S-LFa-R2	S-LFa-P2	S-LFo-R5	S-LFo-R2	S-LFo-P2
-	(8.0%) ^#^	(15.9%) ^#^	(81%) ^#^	(7%) ^#^	(36%) ^#^	(63%) ^#^
ArCHO + ArCOR + ArOH + Ar(CH_2_)_3_OH	-	1.1	22	14	13	31
Ar-COOH + ArCHCHCOOH	-	-	5.3	-	5.6	16
R-COOH + R-COOR’	2.6	2.1	4.1	-	2.2	-
Total monomers	2.6	3.2	31	14	21	47

^#^ Fraction analyzed; Ar: aromatic residue; R: aliphatic chain.

**Table 4:** Summary of the GC/MS results of fractions coming from aqueous and organic processes applied on K-LF-0 illustrating the estimated amount of volatile compounds, divided in three main groups. Data reported as the total mass of compounds/mass of initial fraction (%). Values were rounded up to the nearest two significant figures with an estimated relative error of ± 0.01.

Compounds	K-LFa-R5	K-LFa-R2	K-LFa-P2	K-LFo-R5	K-LFo-R2	K-LFo-P2
-	(21.5%) ^#^	(17%) ^#^	(66%) ^#^	(21%) ^#^	(39%) ^#^	(77%) ^#^
ArCHO + ArCOR + ArOH + Ar(CH_2_)_3_OH	16	-	17	7.5	13	20
Ar-COOH + ArCHCHCOOH	2.8	-	4.6	-	-	1.3
R-COOH + R-COOR’	22	33	22	4.4	4.3	-
Total monomers	41	33	44	12	17	21

^#^ Fraction analyzed; Ar: aromatic residue; R: aliphatic chain.

**Table 5:** Results of the determination of phenolic hydroxyl groups expressed as vanillin equivalents in the lignin fractions recovered from aqueous and organic processes applied on S-LF-0 (expressed in mmol/g) in the dry lignin. Estimated standard errors ±0.2 mmol/g vanillin equivalent (1 σ, from calibration data).

Fraction	Vanillin Equivalent Content (mmol/g)
S-LF-0	3.06
S-LFa-R5	2.91
S-LFa-R2	3.48
S-LFa-P2	3.35
S-LFo-R5	4.38
S-LFo-R2	5.91
S-LFo-P2	6.35

**Table 6:** Results of the determination of phenolic hydroxyl groups expressed as vanillin equivalents in the lignin fractions recovered from aqueous and organic processes applied on K-LF-0 (expressed in mmol/g) in the dry lignin. Estimated standard errors ±0.2 mmol/g vanillin equivalent (1 σ, from calibration data).

Fraction	Vanillin Equivalent Content (mmol/g)
K-LF-0	4.79
K-LFa-R5	2.28
K-LFa-R2	2.99
K-LFa-P2	3.87
K-LFo-R5	5.90
K-LFo-R2	5.80
K-LFo-P2	4.16

**Table 7:** Detailed hydroxyl/carboxyl quantification by ^31^P-NMR for the fractions recovered from aqueous and organic processes applied on S-LF-0.

Fraction	-OH Aliphatic (mmol/g)	-OH Phenolic (mmol/g)	-COOH (mmol/g)
S-LF-0	1.83	3.54	0.92
S-LFa-R5	1.32	2.63	1.33
S-LFa-R2	1.11	3.61	0.9
S-LFa-P2	0.18	3.33	1.26
S-LFo-R5	1.24	4.19	0.09
S-LFo-R2	1.27	4.52	1.29
S-LFo-P2	1.48	5.2	1.52

**Table 8:** Detailed hydroxyl/carboxyl quantification by ^31^P-NMR for the fractions recovered from aqueous and organic processes applied on K-LF-0.

Fraction	-OH Aliphatic (mmol/g)	-OH Phenolic (mmol/g)	-COOH (mmol/g)
K-LF-0	3.31	5.32	0.57
K-LFa-R5	*	*	*
K-LFa-R2	1.39	2.98	0.50
K-LFa-P2	1.11	4.32	1.51
K-LFo-R5	1.64	5.65	0.48
K-LFo-R2	1.10 **	3.50 **	0.45 **
K-LFo-P2	2.52	4.44	0.55

* ^31^P-NMR measurements could not be performed for this fraction due to extensive precipitation of the sample in the conditions used for the analysis; ** possible underestimation of these values due to the limited solubility of the sample in the solvent used for the analysis.
